# Doctors’ Preferences in the Selection of Patients in Online Medical Consultations: An Empirical Study with Doctor–Patient Consultation Data

**DOI:** 10.3390/healthcare10081435

**Published:** 2022-07-30

**Authors:** Yingjie Lu, Qian Wang

**Affiliations:** School of Economics and Management, Beijing University of Chemical Technology, Beijing 100029, China; 17853319403@163.com

**Keywords:** Internet healthcare, online medical consultation, doctor–patient interaction, doctors’ selection of patients, exponential random graph model

## Abstract

Online medical consultation (OMC) allows doctors and patients to communicate with each other in an online synchronous or asynchronous setting. Unlike face-to-face consultations in which doctors are only passively chosen by patients with appointments, doctors engaging in voluntary online consultation have the option of choosing patients they hope to treat when faced with a large number of online questions from patients. It is necessary to characterize doctors’ preferences for patient selection in OMC, which can contribute to their more active participation in OMC services. We proposed to exploit a bipartite graph to describe the doctor–patient interaction and use an exponential random graph model (ERGM) to analyze the doctors’ preferences for patient selection. A total of 1404 doctor–patient consultation data retrieved from an online medical platform in China were used for empirical analysis. It was found that first, mildly ill patients will be prioritized by doctors, but the doctors with more professional experience may be more likely to prefer more severely ill patients. Second, doctors appear to be more willing to provide consultation services to patients from urban areas, but the doctors with more professional experience or from higher-quality hospitals give higher priority to patients from rural and medically underserved areas. Finally, doctors generally prefer asynchronous communication methods such as picture/text consultation, while the doctors with more professional experience may be more willing to communicate with patients via synchronous communication methods, such as voice consultation or video consultation.

## 1. Introduction

### 1.1. Background

Due to the rapid development of the medical and healthcare industry over the past few years and the continuous advancement of Internet technology, Internet healthcare has become an increasingly popular trend in recent years. Especially following the COVID-19 outbreak, there has been a dramatic increase in demand for noncontact consulting [1], which provides a rare opportunity for the development of Internet-based medical consultation services. An increasing number of online medical consultation (OMC) service platforms, such as Haodf.com and WeMed.com, are experiencing rapid growth throughout China.

Doctors serve as the primary providers of OMC services on the platforms [2,3], and their active contributions and provision of high-quality services are instrumental to the sustainable development of OMCs. Unfortunately, in the current situation, doctors do not seem highly motivated to engage in OMC services and are generally less active online [4,5], especially when they are not adequately compensated for their efforts and contributions [6]. Consequently, a significant number of patients using OMC services are unable to receive timely responses to their inquiries. It becomes a challenging and urgent issue for OMC platforms to motivate doctors to participate in the online consultation. Nonetheless, OMC platforms offer some unique advantages, including that online consultation services are not constrained by a specific space or time, making it easier for doctors to take advantage of their fragmented after-hours time to take part in online consultation services [7,8,9], thereby allowing them to build up their online reputation and enhancing their sense of self-worth.

From the doctor’s perspective, what makes OMC so valuable is that it can change the existing patient-centered selection model to a new model in which doctors and patients may select each other. Take for example traditional outpatient appointments, for which patients are usually free to choose hospitals and doctors, but doctors have no choice but to receive the patients with appointments [10]. By contrast, the OMC service model makes it possible for doctors to choose the right patients. When faced with a large number of patients asking various medical questions online, doctors can choose to answer the questions they are interested in based on their clinical expertise. No matter whether a patient specifically requests to consult a certain doctor or the platform assigns the patient’s question to a certain doctor, the doctor has the option to accept or reject the patient’s consultation according to their preference. Personalized selection of patients allows doctors to make full use of their own initiative and professional strengths, which can help improve the efficiency of online consultation and help them realize their self-worth [11]. Consequently, it is imperative that we investigate what factors influence doctors’ behaviors in selecting patients when using OMC services. This helps online platforms precisely match the right patients for doctors, so as to attract more doctors to join the platforms and maintain their long-term retention.

In recent years, many scholars studying Internet healthcare have begun to consider how to better attract doctors to participate in online medical services and explore what factors influence their motivation to do so. It is commonly believed that doctors can gain a variety of online and offline benefits by providing online medical services. Some studies have shown, for example, that doctors can enhance their reputation within a short period of time by sharing their knowledge online, since their information can be quickly and widely disseminated through the Internet [12]. Moreover, some scholars consider the Internet as a new channel for doctors to carry out multisite practices to earn extra income [13,14,15]. However, most of the existing research only regards the Internet as a new tool and medium for providing medical services, without considering that online and offline service models differ in many ways. A very important aspect that is often overlooked is that, unlike the face-to-face consultation model in which doctors are only passively chosen by patients with appointments, online doctors have the option of choosing the patients they wish to treat when they are faced with a large number of online questions from patients. The fact that doctors can choose their patients is an important reason why they are willing to participate in OMC services, but few scholars have explored what types of patients doctors prefer and are then willing to respond to the patients’ questions.

On the basis of the considerations discussed above, the paper suggests that it is necessary to characterize doctors’ preferences for patient selection in OMC, which can contribute to their more active participation in OMC services. We proposed to exploit a bipartite graph to describe the doctor–patient interaction on OMC platforms, and then used an exponential random graph model to analyze the doctors’ preferences for patient selection. Given that a doctor’s decision to select a patient can be affected by various extrinsic and intrinsic factors, this study further suggests that individual characteristics of doctors and other environmental factors such as the working environment will have an important impact on their selection of patients.

Specifically, this study attempted to answer the following two questions:

RQ1: What types of patients do doctors prefer in the context of OMC?

RQ2: What are the differences in the preferences for patient selection among doctors with different individual characteristics and from different types of hospitals?

### 1.2. Research Hypotheses

There is evidence that online consultation helps in reducing waiting times, waiting lists, and unnecessary appointments for patients who were seeking access to specialist outpatient services, resulting in fewer hospital visits and cost savings to the healthcare system [16,17]. However, OMC services remain underutilized by doctors [18]. At the same time, prior research on the adoption of online consultation focused on a variety of online and offline benefits by providing OMC services, but largely overlooked the issue of the intrinsic barriers and facilitators of the online consultation. Among the facilitators for their use, doctors’ concerns about clinical freedom and doctors’ autonomy in choosing or refusing patients are among the most appreciated facilitators of online consultation [19]. However, factors that determine a doctor’s willingness to patient selection are given insufficient attention, and addressing them properly is expected to promote the adoption of OMC services by doctors.

Considering that doctors’ selection of patients in online consultation is based on and limited to the health information systems (HIS), in line with previous studies of technology acceptance in healthcare settings, factors affecting the adoption of health information technology (HIT) need to be considered in order to better understand doctors’ preferences for patient selection on OMC platforms. Some theories and models have been proposed to investigate the facilitators and barriers of HIT adoption by users in existing research proposals. For example, the Technology Acceptance Model (TAM), as one of the most important theoretical models to explain user acceptance of technology and predict usage intentions of technology [20,21], has been proved in recent studies to be an appropriate model to understand doctors’ acceptance of health information technologies [22,23,24]. However, some scholars, in their meta-analytic reviews concerning the application of TAM in healthcare, proposed that the majority of existing TAM studies in healthcare did not consider individual and professional factors, such as doctors’ specialties, advanced skills, and knowledge regarding the use of HIT [22,25,26]. So, we should use extended TAM with more nontechnical considerations to better understand what causes doctors to ignore these systems and explain their attitude toward HIT adoption and use. A systematic literature analysis concerning the barriers and facilitators for the implementation of HIT services was conducted to categorize the factors for HIT adoption into four main categories: technical, individual, environmental, and organizational [2,27,28], which were used in this paper to explain the doctors’ selections of patients in OMCs.

#### 1.2.1. Technical Factors

In contrast to traditional face-to-face consultations, OMCs can take full advantage of the powerful technical capabilities provided by HIS, and offer many advantages. For example, OMC systems enable patients and doctors to communicate in a variety of ways to meet their own individual needs [29]. Additionally, it is possible to overcome the constraints of time and space associated with traditional face-to-face consultation and help alleviate the problem of uneven distribution of medical services through online consultation [30,31]. However, there are also some disadvantages that should be taken into consideration when using OMC services.

First, it was reported by some healthcare providers that providing medical services via telehealth risks losing certain essential information (related to nonverbal feedback and physical examinations) [32,33], which they believed might affect the service quality. For the patients with complex health conditions requiring complex HIS, such as vital-sign monitoring sensors linked to the healthcare provider’s data center for real-time monitoring [34,35], they might encounter technical issues and difficulties because these complex systems are not easy to obtain and use at home [36,37,38]. To date, most physical examinations, laboratory tests, imaging modalities, and operations are not available on the Internet. The clinical information obtained online may be incomplete, resulting in a missed diagnosis or misjudgment. Therefore, many doctors believe that telehealth may not be applicable or appropriate for all sorts of patients, especially for severely ill patients, since incomplete online medical procedures may affect the accuracy of doctors’ judgments about their diseases. To ensure high-quality medical care, doctors preferred those mildly ill patients at low risk of worsening disease due to inappropriate treatment. Therefore, we propose the following hypothesis:

**H1.**  *Mildly ill patients are more likely to be selected by doctors in OMC services.*

Second, the availability of online consultation is not restricted by geographic location, which makes it possible for doctors to serve patients from diverse locations across the country, including urban and rural areas. Especially in the rural and underserved areas, a shortage of doctors is common, and this will dramatically increase in the near future [39]. OMC is considered an important alternative to in-person consultation [40,41]. It enables patients living in underserved areas to access essential healthcare services and eliminates patients’ travel times and travel expenses that are incurred when seeking face-to-face health consultation [42,43,44]. Since patients in remote areas have a more urgent need for online consulting services, they will have a more positive perception of online consultation services and be more willing to cooperate with doctors in online consultation. They will try their best to develop good communication with their doctors to ensure the best-quality online consultation services [45]. Consequently, they appreciate the benefits of doctors’ services and have a higher level of satisfaction with the services they receive from their doctors, thus enabling doctors to gain a great sense of achievement and satisfaction from their work.

Contrary to this, patients in urban areas with abundant healthcare resources have higher expectations for the quality of healthcare they will receive, as well as more options when choosing a healthcare provider. Because of this, even if OMC offers some convenience, it is difficult for them to fully appreciate the value of the service and have a high level of satisfaction with the online services provided by doctors [45] due to a perception that such services are not as satisfying as a face-to-face consultation.

From the doctors’ perspective, the extent of medical resources available to patients, as well as patients’ attitudes and satisfaction with online consultation, play a significant role in doctors’ adoption of online services [46]. Patients from underserved areas will be more likely to be prioritized by doctors, which in turn will lead to greater patient satisfaction and word-of-mouth for their online clinic practice. Therefore, we proposed the following hypothesis:

**H2.**  *Patients from underserved areas are more likely to be selected by doctors in OMC services*.

Finally, it is common practice for OMC platforms to provide different types of synchronous or asynchronous consultation services for both doctors and patients, which allows them to communicate in an easy and convenient manner [9].

Synchronous consultation refers to the delivery of health information in real time. This allows for a live discussion via video or telephone with the patients to deliver medical expertise. Synchronous consultation has the advantage of more efficient communication and a more enjoyable consultation experience for patients, similar to the in-person setting [47,48,49]. However, the disadvantage is that it is rather time-consuming for the doctors, as they must schedule in advance and take a lot of time to hold a one-to-one remote consultation [37,50]. In addition, successful synchronous remote communication will involve consideration of some environmental factors. Having good bandwidth and other high-standard hardware conditions is very important for good-quality real-time communication [38,51]. These factors may further limit synchronous consultation.

Asynchronous consultation uses the “store-and-forward” technique [52], in which patients can send initial medical requests and follow-up photos and videos attached to a description of how they are feeling or how they are recovering to a specialist doctor for diagnostic and treatment expertise, and after that, the attending doctors will make a diagnosis on the basis of information provided by the patients and then reply to the patients with their findings. This has an advantage in that the two parties do not have to be available simultaneously [53,54]. The consultation is carried out without the patient being present at a time convenient to the doctors involved, which helps in reducing waiting times and unnecessary appointments [55]. In addition, there are lower requirements for the technical environment. Doctors are likely to perceive that asynchronous online consultation will improve the productivity of consultation services and enhance the usefulness and ease of use of the consultation. Therefore, we proposed the following hypothesis:

**H3.**  *Patients using asynchronous communication methods such as picture/text consultation are more likely to be prioritized by doctors in OMC services.*

#### 1.2.2. Individual Factors

Existing research points out that individual differences may lead to different optimal behaviors that are driven by specific characteristics [52]. Some recent studies have found that some sociodemographic characteristics such as age, gender, specialty, and years of professional experience may have an impact on doctors’ adoption and use of HIT [2,56]. When doctors engage in OMC services, their main priority is to treat patients based on their professional experience [57], as online consultation makes it difficult for doctors to accurately assess a patient’s condition due to limitations such as the inability to perform physical examinations, etc. In this study, we focused on the professional experience and investigated the effects of doctors’ personal experiences on their selections of patients.

First, according to social cognitive theory, individuals with extensive professional experiences possess a greater sense of self-efficacy, and they tend to be more willing to undertake challenging roles [58]. Thus, doctors with more professional experience are more likely to accept patients with more serious conditions because they believe that they can deal with patients’ health problems more effectively compared to those with less professional experience.

Second, studies have indicated that as a doctor’s experience increases, their clinical behavior is more driven by intrinsic motivation, rather than extrinsic rewards [18,59]. When engaging in online medical services, those doctors who have long professional experience may have higher levels of empathic concern and stronger altruistic values [60], and are more likely to engage in altruistic behaviors. They will view telemedicine as a valuable resource for the delivery of healthcare to patients in underserved areas, as it is known that patients who reside in medically underserved areas are disadvantaged in terms of affordability, accessibility, and availability when seeking healthcare [61]. Those doctors with more professional experience have a greater sense of responsibility to reduce geographic disparities in the healthcare workforce, and are more inclined to help patients from underserved areas.

Finally, the motivations for participating in online consultation are different for different doctors [62]. The aim of doctors who have less professional experience may be to gain practical experience and increase their popularity. Those who have a high level of expertise, however, care more about maintaining their online reputation through online consultation [63]. They wish to be able to communicate effectively with patients and maintain a positive doctor–patient relationship in order to be rated highly by their patients. Due to this reason, when facing many means of online consultation available, they will be more willing to communicate with patients via synchronous communication methods such as telephone consultation or video consultation. Real-time communication may be able to help patients gain more emotional support, improve their experiences in communicating with their doctors, and increase their trust in their doctors, thereby enabling the doctors to obtain positive online word-of-mouth.

To sum up, the following assumptions were made:

**H4a.**  *The doctors with higher professional experience are more likely to prefer severely ill patients*.

**H4b.**  *The doctors with higher professional experience are more likely to prefer patients from underserved areas*.

**H4c.**  *The doctors with higher professional experience are more likely to communicate with the patients using synchronous communication methods such as telephone consultation or video consultation*.

#### 1.2.3. Environmental and Organizational Factors

Existing theories on the use of HIT, represented by the theory of planned behavior (TPB), state that HIT adoption and use is not completely a result of users’ own free choices. The external environment is one of the factors that indirectly influences their intentions, and as a result, their actual behavior [52]. In the context of OMCs, doctors’ selections of patients is also significantly influenced by the external environment and organization; i.e., the hospitals where they work. It is generally believed that higher-quality hospitals can attract more demand and raise revenues, whereas those with poor quality may lose revenues [45], particularly in those national healthcare systems in which citizens are entitled to freely choose any public or private providers in the country. Thus, healthcare providers have strong incentives to produce high-quality care. The quality of healthcare is multifaceted, including clinical quality, patient experience (such as being treated with respect and being able to communicate and have a dialogue with the doctor), and availability of services (such as how long patients need to wait for healthcare) [64]. When participating in Internet healthcare services, doctors affiliated with high-quality hospitals are expected to make full use of their advantages to attract potential patients who are likely to benefit more from OMC services and provide such patients with high-quality services.

First, it is generally believed that low-quality hospitals have a relatively higher mortality rate, higher hospital-acquired infection rates, and worse patient perceptions of their care [64]; therefore, severely ill patients with a considerable risk of mortality may be more sensitive to the quality of medical care delivered by doctors and hospitals [65]. They are more likely to require the medical services provided by hospitals with a high quality, since high-quality hospitals had an observed mortality rate and other outcomes that were better than expected. Because patients’ preferences for types of hospitals vary across disease severity, for the doctors affiliated with high-quality hospitals, selecting patients who are critically ill may have a greater marginal effect on improving patients’ perception of online-service quality offered by their hospitals [65]. In addition, theories of health provider behaviors suggest that providers are motivated not only by their own benefits, but also by an altruistic interest in the health of their patients [66]. Altruistic behavior may be motivated by multiple factors, in which professional ethical standards may play a significant role in motivating altruistic behavior among health providers. Therefore, when critically ill patients participating in an Internet medical consultation are expected to receive higher-quality medical care, the doctors from high-quality hospitals will feel more obligated to meet their needs.

Second, the patients who live in rural or underserved areas may have a greater need for high-quality hospitals, because the hospitals located in lower-income neighborhoods or rural areas may be perceived to have lower-quality care, and thereby have fewer patient visits and admissions [45]. In spite of this, it is often difficult for these patients to bypass the surrounding hospitals and travel to the larger hospitals, as the most important hospitals with a high quality are predominantly located where the population and economic activities are more concentrated [67]. They often have to make a trade-off between hospital quality and travel cost, resulting in a significant share of patients unwilling to accept additional travel time to obtain treatment in a hospital with a better reputation [68]. Under these circumstances, online medical services provide a viable alternative for patients in underserved areas who wish to visit large hospitals but have difficulty doing so. We have reason to believe that when receiving online consultation requests from patients in different regions, doctors from high-quality hospitals are more likely to choose patients in underserved areas for altruistic reasons.

Finally, providing a timely response to patients’ queries is considered an important factor in the quality of medical care. Results show that patients with higher waiting times were less satisfied with healthcare quality, as long pretreatment waiting times may allow the condition to worsen or even increase the risk of mortality [45]. In a telemedical consultation, asynchronous consultation has the advantage that the two parties do not have to be available simultaneously. Doctors can respond to patients’ health concerns as soon as possible whenever consultation is required, which helps to avoid unnecessary appointments and reduces appointment waiting times [69]. Compared to synchronous consultation, asynchronous consultation will result in the patient’s perception of their waiting time to see the doctor to be “shorter than expected”, thus enhancing patients’ satisfaction with consultation services [70]. The benefits of rapid response through asynchronous communication will no doubt help hospitals build their online reputations and thus attract more patients, even though the quality of medical care does not seem to be superior to others. Therefore, we inferred that asynchronous consultation is more likely to be preferred by doctors from high-quality hospitals and is expected to improve the perceived quality of their hospitals.

To sum up, the following assumptions are made:

**H5a.**  *The doctors from high-quality hospitals are more likely to prefer severely ill patients*.

**H5b.**  *The doctors from high-quality hospitals are more likely to prefer patients in underserved areas*.

**H5c.**  *The doctors from high-quality hospitals are more likely to communicate with their patients via asynchronous communication*.

## 2. Materials and Methods

### 2.1. Data

We chose a popular online medical platform that offers OMC services in China as our data source. There are more than 240,000 registered doctors on this platform, providing medical services to patients in more than 72 million visits. The most frequently consulted diseases in OMC services were reproductive system diseases (17.31%), skin diseases (15.82%), digestive system diseases (13.33%), nervous system diseases (9.96%), respiratory diseases (8.58%), endocrine system diseases (8.25%), motor system diseases (7.75%), circulatory system diseases (5.65%), psychological disorders (4.91%), urinary system diseases (4.73%), and immune system diseases (3.71%).

We focused on chronic diseases because they require long-term treatment. Patients need to communicate with their doctors regularly over a long period of time for self-management of their health [71]. Compared with patients with other diseases, patients with chronic diseases were more suitable to communicate with doctors through OMC services, as proved by the fact that chronic diseases are one of the most frequently consulted diseases in OMC services, and account for up to 58.07%. Among the many chronic diseases, hypertension and diabetes were chosen because they represent a relatively high proportion of the chronic disease population and are widely distributed in all regions of China. These two diseases are also frequently selected as representative diseases in many studies related to chronic diseases.

The platform provides open data related to OMC services for researchers in health-related fields. After the application was approved, we were given access to the dataset related to OMC services. The dataset stores detailed information on OMCs offered on the platform. The available data in the dataset included consultation-related information (consultation time, consultation fees, communication type, etc.), patient-related attribute information (type of diseases, disease severity, residence location, etc.), and doctor-related attribute information (professional titles, affiliated hospitals, etc.). It is worth noting that we only used data from free consultation services and excluded data from paid consultation services to avoid the influence of material interests on the selection of patients. Therefore, we obtained a sample consisting of 1404 doctor–patient consultation data from the OMC platform for empirical analysis.

The five variables we chose are shown in Table 1. Patient-related attributes included disease severity, the availability of medical resources, and the type of communication. (1) Disease severity was divided into two types: mildly ill and severely ill. The severity of the patient’s illness was determined by the OMC service platform according to the patient’s self-described information related to the disease. While a patient fills in the information necessary for the diagnosis of the disease, including personal symptoms, medical history, previous hospital visits, and clinical reports, the OMC system will assess the severity of the disease according to the self-described conditions and then assign the severity level. We found that 83.86% of the population was mildly ill patients and only 16.14% of the population was severely ill patients. (2) The availability of medical resources was divided into three levels: low, medium, and high. There have been many studies in recent years that analyzed the distribution of medical resources in China. According to the literature [72], we obtained demographic, economic, and geographic area data from the *China Statistical Yearbook* and the data related to medical resources and medical services from the *China Health Statistics Yearbook*. In addition, the distribution of high-quality healthcare resources in China was taken into consideration according to the literature [73]. We then conducted a comprehensive evaluation of the distribution of medical resources across locations based on the metrics proposed in the literature [72] and classified all regions into three groups. We found that 15.97% of the patients lived in areas with low medical resources, 70.42% of the patients lived in areas with medium medical resources, and 13.61% of the patients lived in areas with high medical resources. (3) The type of communication used by patients included synchronous consultation (e.g., telephone consultation) and asynchronous consultation (e.g., picture/text consultation). Doctor-related attributes included their professional experience and the quality of hospitals where they worked. We found that 6.11% of the patients preferred to use synchronous consultation and 93.89% of the patients prefer to use asynchronous consultation. (4) According to the literature [74], there is a professional title evaluation system in the context of Chinese healthcare systems. The professional ranking of doctors can be divided into four tiers, from low to high: resident physician, attending physician, associate chief physician, and chief physician. Doctors with higher professional titles have more medical knowledge and longer clinical experience. So, in this study, we considered officially certified chief physicians and associate chief physicians as experienced doctors, and the others as doctors with low professional experience. We found that 36.19% of the population was doctors with low professional experience, and 63.81% of the population was doctors with high professional experience. (5) According to the literature [75], hospitals in China are organized in a 3-tier system that recognizes their abilities to offer medical care, provide medical education, and conduct medical research. Class 1 hospitals (or primary hospitals) are the hospitals and health centers that provide preventive care and basic healthcare and rehabilitation services to local communities. Class 2 hospitals (or secondary hospitals) are regional hospitals that offer comprehensive medical and healthcare services to multiple communities, provide medical education, and conduct medical research. Class 3 hospitals (or tertiary hospitals) are regional hospitals that provide specialist medical and healthcare services and carry out high levels of teaching and scientific research tasks. So, in this study, we considered officially certified tertiary hospitals as high-quality hospitals and the others as low-quality hospitals. We found that 23.64% of the doctors worked in low-quality hospitals and 76.36% of the doctors worked in high-quality hospitals.

**Figure 1:**
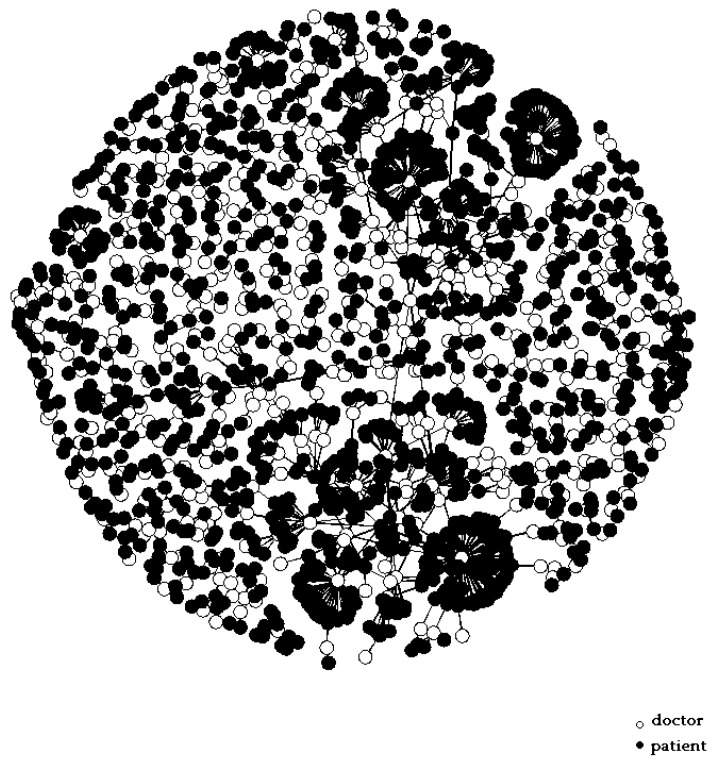
Bipartite graph network of doctor–patient consultations.

**Figure 2:**
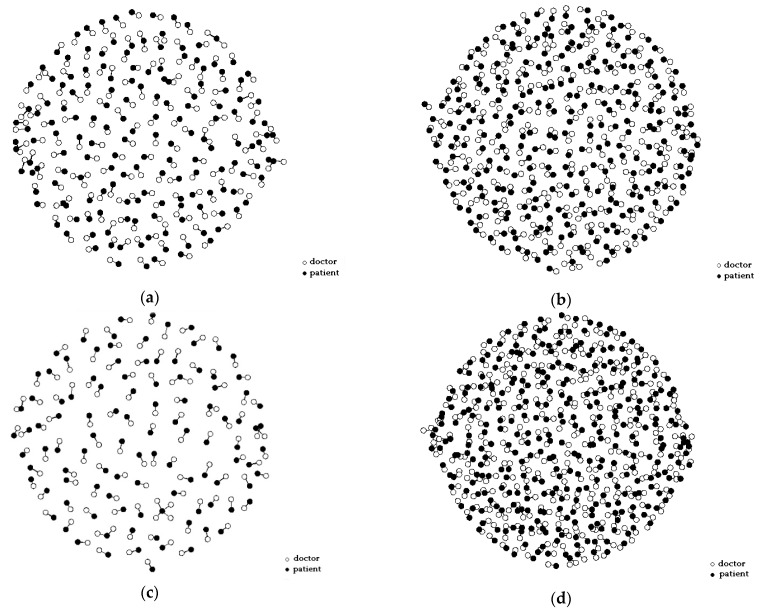
Separate bipartite graph networks for doctors with different professional experience and from different types of hospitals: (**a**) Doctors with low professional experience; (**b**) Doctors with high professional experience; (**c**) Doctors from low-quality hospitals; (**d**) Doctors from high-quality hospitals.

The bipartite graph network was constructed using doctor–patient consultation data, as shown in Figure 1. Patients are represented by the black dots and doctors are represented by the white dots. The edges connecting two nodes represent the interaction between doctors and patients. The graph reveals that some doctors selected a large number of patients, indicating that the number of patients treated by doctors was unevenly distributed. Figure 2 shows the bipartite graph networks constructed separately for the doctors with different professional experience and from different types of hospitals. It can be seen that the doctors with high professional experience or working in high-quality hospitals made up a larger proportion of the population, and they provided OMC services to a greater number of patients than other groups. This shows that there was a Matthew effect in the network The Matthew effect is described as the phenomenon in societies in which the rich tend to become richer and the potent even more powerful. It is closely related to the concept of preferential attachment in network science, in which the more connected nodes are destined to acquire many more links in the future than the auxiliary nodes [76].

### 2.2. Method

This study used an exponential random graph model (ERGM) to analyze the doctors’ preferences for patient selection. The ERGM is a statistical model used to analyze network formation. The general form of the bipartite ERGM is shown in Equation (1) [77,78], where X is the space of stochastic adjacency matrixes that contains all possible relationships between nodes in the network; x is a particular instance of the stochastic adjacency matrix—here, a matrix of actual zeros and ones; and θ is a collection that contains the parameters of all network configurations. Using Equation (1) with the given parameter θ will assign a probability to x based on the number of network configurations; z_k_(x)(k = 1, 2, …, p) are counts of configurations in the graph x, θ_k_(k = 1, 2, …, p) weight the relative importance of their respective configurations, and the normalizing term k(θ) ensures that the sum of the probability mass function (P_θ_(x)) is one.

Pr(X = x|θ) = 1/k(θ) exp{θ_1_z_1_(x) + θ_2_z_2_(x) + … + θ_p_z_p_(x)} (1)

Our bipartite ERGM included thirteen parameters: two parameters for purely structural effects (Edges and Alternating K-Stars), five parameters for actor–relation effects (Professional Expertise, Hospital Quality, Disease Severity, Medical Resources, and Communication Type), and six parameters for actor–relation interaction effects. To better understand how to use bipartite ERGMs to test our hypothesis, we have provided a graphical presentation of all types of effects used in the model, along with the corresponding research hypotheses, in Table 2.

## 3. Results

We estimated the ERGMs using Markov chain Monte Carlo maximum likelihood estimation (MCMC-MLE) methods and implemented the simulation-based algorithms for MCMC-MLE in the statnet software suite developed for the R platform. Table 3 presents the results of the ERGM estimates for the bipartite network. According to the literature [79], a positive (negative) parameter in the results of ERGM estimates indicates that nodes with the characteristic tend to have higher (lower) network activity or more (less) ties than nodes without the characteristic, or that a corresponding configuration is more (less) likely to occur.

First, we examined actor–relation effects for patient-related attributes. A negative and significant estimate for the DS_Severely_ill parameter indicated that mildly ill patients had a higher likelihood of being selected by doctors. Thus, H1 was supported. The positive and significant estimates for MR_Medium and MR_High indicated that, compared with patients in areas with low medical resources, doctors generally preferred to choose patients in areas with medium and high medical resources. Hence, H2 was not supported. A positive and significant estimate for CT_Asynchronous indicated that, compared with synchronous consultation such as telephone consultation, doctors were more inclined to choose patients who used asynchronous consultation, such as picture/text consultation. Thus, H3 was supported.

Second, we examined the interaction effects between patient-related attributes and doctor-related attributes. To investigate the effects of doctors’ professional experience on their selections of patients, we examined the estimates of the following parameters: PE_High*DS_Severely_ill, PE_High*MR_Medium, PE_High*MR_High, and PE_High *CT_Synchronous. A positive and significant estimate for PE_High*DS_Severely_ill indicated that doctors with higher professional experiences were more inclined to choose severely ill patients; thus, H4a was supported. The negative estimates for PE_High*MR_Medium and PE_High*MR_High indicated that when doctors with higher professional experience chose patients in areas with different medical resources, they were more inclined to choose patients in areas with weak medical resources. Therefore, H4b was supported. A positive and significant estimate for PE_High*CT_Synchronous indicated that doctors with higher professional experiences preferred patients who used synchronous consultation such as telephone consultation; thus, H4c was supported. To investigate the effects of hospital quality on doctors’ selections of patients, we needed to examine the estimates of the following parameters: HQ_High*DS_Severely_ill, HQ_High*MR_Low, HQ_High*MR_Medium, and HQ_High*CT_Asynchronous. The parameter HQ_High*DS_Severely_ill was not significant, indicating that doctors from high-quality hospitals had no significant preference for patients with different disease severities. Hence, H5a was not supported. The positive and significant estimates for HQ_High*MR_Low and HQ_High*MR_Medium indicated that doctors from high-quality hospitals were more inclined to choose patients in medically underserved areas, thus supporting the null hypothesis H5b. The parameter HQ_High*CT_Asynchronous was not significant, indicating that doctors from high-quality hospitals had no significant preference for the patients using different means of consultation. Hence, H5.z was not supported.

**Figure 3:**
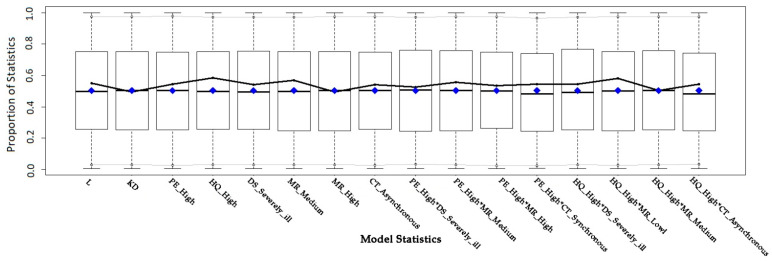
GOF test of the bipartite ERGM.

The goodness-of-fit (GOF) statistics, which were calculated for the bipartite ERGM, allowed us to know whether the specified model for our observed data represented particular network configurations or graph features well. The results of the GOF test are shown in Figure 3. The 16 boxplots in Figure 3 respectively represent the range of the proportions of 16 configurations that made up the ERGM model in the simulated network. The black line in the middle represents the quantiles of the 16 configurations of the observed network in the simulated networks [79]. In a good fit, the observed statistics should be near the sample median (0.5). As seen in Figure 3, the proposed model fit the data well.

## 4. Discussion

Our results suggested that doctors were found to have significant preferences for their selection of patients in the context of OMC services. Firstly, from the perspective of disease severity, doctors generally preferred to select patients with mild symptoms. The lack of physical examinations and poor online communication performance can make it difficult to ensure the accuracy of a doctor’s diagnosis [33]. Therefore, it is a reasonable choice for most doctors to select patients with minor illnesses to better avoid the risk of misdiagnosis. Secondly, from the perspective of the geographical area where the patient lived, the study found that doctors were generally more inclined to choose patients in areas with medium and high medical resources, rather than the patients in areas with weak medical resources, as mentioned in one of our hypotheses. One of the possible explanations is that online doctors may consider that patients in areas with medium and high medical resources are likely to transfer to offline hospitals where the patients’ require further examination and treatment after receiving online consultation services [80]. Therefore, to improve the conversion rate of online users to offline visitors, doctors may choose patients who are in the same geographic area as them, rather than in remote and medically underdeveloped areas. Finally, from the perspective of the means of doctor–patient consultation provided by the platform, doctors were generally more inclined to communicate with patients using asynchronous consultation such as picture/text consultation. This was mainly because many doctors have limited time and energy, and thus must devote their leisure time to dealing with online consultation, which makes it difficult for them to have real-time communication with their patients. In addition, asynchronous communication allows doctors to read or respond to messages at a time convenient to them, and thereby improves the efficiency of OMC services.

We further discussed the impact of doctors’ individual characteristics on their selections of patients in OMC services. This study mainly focused on the doctors’ professional experience and analyzed the differences in the choice of patients among doctors with different professional experience. First of all, doctors with high professional experience tend to choose more severely ill patients, because they feel more morally responsible for critically ill patients who desperately need their help, and are subsequently more likely to give priority to these patients in OMC services. Secondly, we found that doctors with high professional experience paid more attention to the patients in areas with weak medical resources. Similar to the reasoning above, doctors with high professional experience may feel more morally responsible for patients in medically underserved areas who desperately need their help, and are subsequently more likely to give priority to such patients in OMC services. Finally, doctors with higher professional experience are more willing to communicate with patients via synchronous communication methods such as telephone consultations. Even though asynchronous consultations are more time-consuming, they allow doctors to communicate with patients more efficiently and effectively, in turn leading to increased patient satisfaction and a better doctor–patient relationship, which is especially important for doctors with higher professional experiences [63].

Finally, we discussed the impact of environmental and organizational factors on doctors’ selections of patients in OMC services. We focused on the quality of hospitals where doctors work and analyzed the impact of hospital quality on doctors’ selections. A valuable finding was that the doctors from high-quality hospitals gave priority to the patients in medically underserved areas when faced with a large number of online questions from patients. One reasonable explanation is that patients in underserved areas have little access to quality medical care and are therefore more eager to have good online communication with their doctors, and their satisfaction is relatively high [45]. This in turn motivates doctors to interact with these patients in order to improve their online reputation.

## 5. Conclusions

This study focused on doctors’ preferences for patient selection in the context of OMC services and investigated which types of patients doctors preferred to interact with, and then examined the differences in the preferences for patient selection among doctors with different individual characteristics and affiliated hospitals.

This study offered important theoretical contributions in the area of OMCs. Researchers have conducted a significant amount of research to explore what doctors should do to obtain more online and offline benefits, such as increasing the quantity and improving the quality of OMC services. However, few scholars considered that online doctors have the option of choosing the patients they wish to treat, and thereby explored what types of patients doctors preferred and were then willing to respond to their questions. We developed a new theoretical model based on TAM, and proposed that technical, individual, environmental, and organizational factors would have significant effects on the doctors’ selections of patients in OMCs. Furthermore, our research extended this work by considering the differences in doctors’ professional experience and the hospitals where they work and investigating their effects on their selection behaviors. The empirical results revealed that some of the hypotheses were supported, whereas others were not.

Our findings also had some important practical implications. First, our empirical results can help OMC platform managers better understand what factors influence doctors’ behavior in selecting patients when using OMC services. This will help online platforms precisely match the right patients to doctors, so as to attract more doctors to join the platforms and maintain their long-term retention. Second, the results revealed that some environmental and organizational factors had significant effects on the doctors’ selections of patients in OMCs. Thus, policymakers should take specific measures to encourage large high-quality public hospitals to support those medically and socioeconomically disadvantaged patients and ensure equitable access to healthcare for diverse populations. For example, Beijing, as the capital of China, has a large number of high-quality hospitals. Our research may help these hospitals to better serve patients in remote areas through Internet healthcare, thus effectively alleviating the problems of uneven distribution of medical resources in China.

This study had some limitations. First, we were limited to the data retrieved from a popular online healthcare platform in China. More OMC data from other healthcare systems or other countries are needed to test whether the results are consistent with our findings. Second, we identified some individual factors that had significant effects on doctors’ selections of patients. However, there are still some other factors, such as some demographic characteristics including gender, age, and income, that played important roles in their selection behaviors but were not uncovered in our study. These additional variables may be incorporated into the models in future studies.

## Figures and Tables

**Table 1:** Variable description.

	Variable	Description	Percentage
Patient-Related Attributes	Disease severity (DS)	0 = “Mildly ill”	83.86%
		1 = “Severely ill”	16.14%
	Medical resources (MR)	1 = “Low”	15.97%
		2 = “Medium”	70.42%
		3 = “High”	13.61%
	Communication type (CT)	1 = “Synchronous”	6.11%
		2 = “Asynchronous”	93.89%
Doctor-related Attributes	Professional experiences (PE)	0 = “Low”	36.19%
		1 = “High”	63.81%
	Hospital quality (HQ)	0 = “Low”	23.64%
		1 = “High”	76.36%

**Table 2:** Summary of network configurations included in the bipartite ERGM.

Parameter	Figure	Hypothesis	Description
Edges (L)	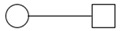	_	Baseline probability of forming a tie between a doctor and a patient
Alternating K-Stars for Doctors (KD)	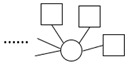	_	Measures of the degree distributions of doctor nodes
Professional Expertise (PE)	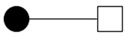	_	Doctors with high professional experience have a higher likelihood of selecting patients
Hospital Quality (HQ)	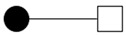	_	Doctors from high-quality hospitals have a higher likelihood of selecting patients
Disease Severity (DS)	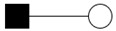	H1	Severely ill patients have a higher likelihood of being selected by doctors
Medical Resources (MR)	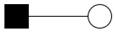	H2	Patients in areas with medium or high medical resources have a higher likelihood of being selected by doctors
Communication Type (CT)	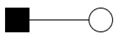	H3	Patients using asynchronous consultation have a higher likelihood of being selected by doctors
PE*DS	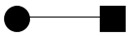	H4a	Doctors with high professional experience are more willing to choose severely ill patients
PE*MR	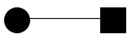	H4b	Doctors with high professional experience are more willing to choose patients in areas with medium or high medical resources
PE*CT	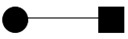	H4c	Doctors with high professional experience are more willing to choose patients using synchronous consultation
HQ*DS	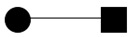	H5a	Doctors from high-quality hospitals are more willing to choose severely ill patients
HQ*MR	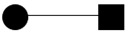	H5b	Doctors from high-quality hospitals are more willing to choose patients in areas with low or medium medical resources
HQ*CT	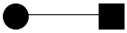	H5c	Doctors from high-quality hospitals are more willing to choose patients using asynchronous consultation

Note: boxes represent patients, black boxes represent patients with certain attributes, circles represent doctors, and black circles represent doctors with certain attributes. If doctor i selected patient j, x_ij_ = 1; otherwise, x_ij_ = 0.

**Table 3:** Parameter estimates for the bipartite ERGM.

Parameter	Estimates	*p* Value
L	−7.27179	0.000 ***
KD	0.42970	0.081.
PE_High	0.47885	0.003 **
HQ_High	−0.23438	0.448
DS_Severely_ill	−0.80399	0.000 ***
MR_Medium	0.39400	0.039 *
MR_High	0.55863	0.018 *^,^^†^
CT_Asynchronous	0.90550	0.013 *
PE_High*DS_Severely_ill	1.25818	0.000 ***
PE_High*MR_Medium	−0.49638	0.004 **
PE_High*MR_High	−0.25364	0.251
PE_High*CT_Synchronous	0.80215	0.012 *
HQ_High*DS_Severely_ill	−0.03456	0.827
HQ_High*MR_Low	0.44126	0.034 *
HQ_High*MR_Medium	0.43532	0.011 *
HQ_High*CT_Asynchronous	−0.32621	0.254

Note: ‘***’, ‘**’, ‘*’, and ‘^†^’ represent *p* < 0.001, *p* < 0.01, *p* < 0.05, and *p* < 0.1, respectively.

## Data Availability

The data used to support the findings of this study are available from the corresponding author upon request. The platform on which the information was collected has been anonymized. All data have been desensitized, and there was no content that presented privacy issues for any of the related parties.
